# The Treatment of Organic Urethral Stricture

**Published:** 1890-01

**Authors:** F. W. McRae

**Affiliations:** Atlanta, Ga.


### THE TREATMENT OF ORGANIC URETHRAL STRICTURE.

BY F. W. McRAE, M. D., ATLANTA, GA.

The definitions of stricture as given in the text-books are vague and inaccurate, including many conditions which we do not recognize as stricture. By a general consensus of opinion, however, the term is limited to an unnatural narrowing of the urethral caliber, due to pathological changes in the mucosa and peri-urethral tissues. No amount of simple inflammatory thickening of the mucous membrane is sufficient to constitute stricture, or produce retention of urine in a previously normal urethra. There must be a peculiar change in the mucous membrane rendering it more or less pervious and permitting urinary infiltration into the peri-urethral structure, a condition which has been very clearly described by Dr. Jno. P. Bryson, in a paper read before the American Association of Genito-Urinary Surgeons, May 21, 1889, in which he suggests the name, "chronic contracting peri-urethritis," as better expressing the pathological condition.

Dr. Reginald Harrison, of Liverpool, in his Laettesomian lectures, 1888, was the first surgeon to clearly demonstrate the etiological factor of primary changes in the mucosa as the "essential though passive element in the production of stricture." Without that peculiar change, no matter what degree of inflammation may exist, stricture is never produced. When, however, that change has occurred, "urine leakage" takes place and stricture is the consequence.

The adventitious tissue developed as the result of urinary infiltration is the active factor in the production of stricture. This new tissue, the characteristic feature of which is a tendency to indefinitely contract, continues to be developed so long as leakage exists, and the development seems to be a conservative attempt on the part of nature at limiting the extension of the disease and preventing the formation of urinary fistulæ.

Were it not for this process, we would have active and extensive inflammation, with the serious consequences so frequently observed to follow trauma of the urethra, instead of a slowly progressing peri-urethritis.

Bryson, in a paper already alluded to, has demonstrated the existence of a dilated condition in front of as well as behind the strictures. He accounts for this state of affairs by supposing the mocous membrane to have lost its natural elasticity as the result of the previous inflammation.

That stricture is the result of the changes above alluded to, seems to me to have been fully demonstated and entirely consistent with the clinical history and pathological anatomy.

In this paper it has not been my intention to enter into an exhaustive consideration of the etiology of stricture, but simply to present the more prominent facts, a knowledge of which is essen tial to a rational treatment of the condition. I have alluded briefly to the essential etiological factors, without discussing at all the various inflammatory conditions capable of bringing about that change in the mucous membrane, which is the beginning of organic stricture. Nor have I made any allusion to congenital narrowings of the urethra, as they do not come within the scope of this paper. By far the larger number of strictures are found anterior to the cut-off muscle. In character they are, (1) soft, according to Keyes; (2) simple fibrous; (3) inodular.

The soft stricture is often met clinically, and is capable of causing retention though there be but slight organic change. The retention in these cases is due to spasm induced by the constant irritation. The spasm is often so intense as to prevent the introduction of a filiform bougie, while a larger instrument can generally be passed with comparative facility. They are generally very readily cured by rapid dilatation, though some prove quite rebellious. That strictures of the second class, or simple fibrous variety, situated in the spungy urethra, may be permanently cured has been abundantly demonstrated. To Dr. Otis all credit is due for the brilliant results obtained by dilating internal urethrotomy.

That this class of strictures may be cured by gradual dilatation, I think, is unquestioned—that they usually are not is generally conceded. The length of time required to effect a cure is so great in strictures of small caliber that few patients are willing to give the treatment the necessary time and care. I have little faith in electrolysis. The method of treatment which I prefer is dilating internal urethrotomy with Dr. Otis' dilating urethratome. Where the stricture is of small caliber a certain amount of dilatation must be obtained before the instrument can be used. This, however, is generally very readily accomplished. The operation, with the proper attention to detail, and aseptically performed, is almost if not quite as safe as gradual dilatation. Otis has done the operation hundreds of times without a fatal result. Other operators have obtained equally good results. Under cocaine anæsthesia the operation is painless.

Before operating I always immerse all the instruments which I may need in a three per cent. solution of carbolic acid for at least half an hour preceding the operation, so as to render them thoroughly aseptic. I next thoroughly cleanse with a stiff brush, soap and water all the parts of the patient with which my hands or instruments must come in contact, and disinfect them with a $\frac{1}{1000}$ solution of bichloride of mercury. In other words I prepare the parts just as I would for an amputation.

I always make a careful examination of the urine before any operation within the urethra.

When there is a urethral discharge, I wash out the urethra with a warm saturated solution of boric acid. The instruments are taken out of the carbolic solution and dipped in boiled water or Thiersch's solution, to remove the acid before introduction into the urethra. I then perform the operation in the usual way, first ascertaining the extent and location of the stricture and the normal caliber of urethra. I always, except in strictures of the first inch, cut the roof of the urethra up to the full size of its normal caliber, as indicated by the urethra-meter. All constricting bands should be cut. The after-treatment consists of the introduction of bougies every third or fourth day, with saline laxatives and diuretics, as indicated, to keep the bowels well open and the urine bland and unirritating. The after-introduction of sounds is an essential part of the treatment, for it allows the cut to fill up from the bottom with granulation tissue instead of simple adhesion of the cut surfaces, which would otherwise occur. This granulation tissue is finally covered with epithelium, and the cure is complete.

REPORTS OF CASES.

CASE I.—W. W., single, *æt.* 28, white, robust physique. Had gonorrhœa three years previously, lasting for several months. Complained of pain on micturition, inability to completely empty urethra, lessening of stream, etc. On examination I found two tough fibrous strictures, one just posterior to fossa navicularis, other two and one-half inches from meatus. Both strictures were cut up to 33, the anterior one on the floor of the urethra with meatatome, the posterior one with urethratome on roof of urethra. These strictures were associated with intense spasm of the cut off muscle and excessive hyperæsthesia of the urethra. The patient was cured without an unfavorable symptom. From a thorough examination made several months after treatment was suspended, I am satisfied the cure is permanent.

CASE II.—W. I., single, *æt.* 28 years, white, nervous temperament. History of gonorrhœa six years previous, lasting eighteen months to two years. Typical history of stricture. On examination, found small meatus, admitting twenty French, and three tight organic strictures; one just posterior to fossa nav., another two and one-half inches from meatus, and a third just anterior to bulb. Cut up to 33 French. After-treatment same as Case I. Have examined patient several times since treatment was stopped, and there is no tendency to recontract, a full sized sound passing in without obstruction.

CASE III.—A. F., single, *æt.* 22 years, white, chronic gonorrhœa, with recurrent exacerbations extending over a period of three years. Examination revealed a peculiar hypospadic malformation. On superficial inspection the meatus seemed to be normal, but on looking closely I found what at first appeared to be the meatus was only a slit about a line in depth, while the opening was underneath the glands just in front of frænum. After slitting up meatus I detected a tortuous stricture occupying the anterior four inches of the canal, and cut it freely up to 34 French. The discharge from the urethra was considerably increased by the operation, but subsequently ceased entirely. The patient, who was a physician, was under my care only three weeks, preferring to conduct the after-treatment himself, as his home was out of the city. I have had several letters from him since he passed out of my hands, which seem to indicate a complete cure.

CASE IV.—Married, white, *æt.* 30. History of gonorrhœa ten years before, lasting several months. Gonorrhœa was cured with difficulty. Gradual diminution of stream with pain and other symptoms of stricture. Very small meatus, admitting 15

French. After incision of meatus I found two very tough fibrous strictures of almost cartilaginous consistency, situated respectively one and one-half and four inches from meatus. The strictures were so tough that it required very considerable force and several incisions before the constrictions were thoroughly relieved. With the exception of persistent hemorrhage and extravasation of blood, the patient made an excellent recovery. I might report a number of other less tedious cases, but as there are no features of especial interest, I will not do so. The cases reported were strictures of very tough fibrous, almost cartilaginous consistency.

Similar cases treated by gradual dilatation have not terminated so favorably. I do not believe I have permanently cured a single fibrous stricture by this method of treatment.

Strictures of the membranous urethra are seldom radically cured by any method of treatment. Here internal urethrotomy is never advisable, on account of the great danger of hemorrhage and the impossibility of cutting with any degree of accuracy. Divulsion is unsurgical and dangerous. Here we must, in the majority of cases, content ourselves with gradual dilatation up to as near the normal caliber of the urethra as possible, and then the occasional introduction of proper sized instruments indefinitely. External urethrotomy offers somewhat better results, not a few being permanently relieved by the operation or by a combination of the external and internal operations. I have treated all the cases that have fallen in my hands by gradual dilatation. In none of the cases have I been able to effect a radical cure except, perhaps, in one case of soft stricture.

The deep inodular strictures are not curable by either of above methods. Only relative relief of the symptoms need be expected.

The operations of excision, with transplantation of healthy mucous membrane, or excision of the entire stricture and suturing together the ends of the severed urethra, have been successfully performed. Theoretically the operations seem to meet the indications. The results obtained thus far apparently warrant the performance of the operations in appropriate cases.

Dr. E. L. Keyes, in a paper read before the American Association of Genito-Urinary Surgeons at New Port, May 21st, and published in the *New York Medical Record* of May 25th, 1889, reviews the operations.

The indications to be met in the treatment of urethral stricture are, (1) to set up an active inflammation in the place of the chronic inflammation which always exists in the stricture; (2) to restore the mucous membrane to its original condition.

An operation which would fully meet these indications would be an ideal operation. We can, with our present methods of treatment, only approximately restore the tissues to their normal condition.

